# Biomarkers in Ataxia-Telangiectasia: a Systematic Review

**DOI:** 10.1007/s00415-024-12766-7

**Published:** 2025-01-15

**Authors:** M. Y. Tiet, B.-I. Guțu, P. Springall-Jeggo, D. Coman, M. Willemsen, N. Van Os, M. Doria, H. Donath, R. Schubert, R. A. Dineen, S. Biagiotti, A. P. Prayle, A. T. Biomarker Working Group, A. E. Hensiek, R. Horvath

**Affiliations:** 1 Department of Clinical Neurosciences, John Van Geest Centre for Brain Repair, University of Cambridge, Robinson Way, Cambridge, CB2 0PY UK; 2 Ataxia Telangiectasia Society, Harpenden, UK; 3 Queensland Children’s Hospital, 501 Stanley Street, South Brisbane, Australia; 4 Department of Pediatrics, Pediatric Neurology, Radboud University Medical Centre, Amalia Children’s Hospital, Nijmegen, Netherlands; 5 Primary Immunodeficiency Unit, Bambino Gesù Children’s Hospital, Rome, Italy; 6 Division of Pneumology, Allergology, Infectiology and Gastroenterology, Department of Children and Adolescent Medicine, University Hospital, Goethe University, Frankfurt, Germany; 7 Mental Health and Clinical Neuroscience, School of Medicine, University of Nottingham, Nottingham, UK; 8 NIHR Nottingham Biomedical Research Centre, Nottingham, UK; 9 Department of Biomolecular Sciences, University of Urbino, Urbino, Italy; 10 Division of Child Health, Obstetrics and Gynaecology, University of Nottingham, Nottingham, UK

**Keywords:** Ataxia-Telangiectasia, Biomarkers, Neurodegeneration, Clinical trials

## Abstract

Ataxia-Telangiectasia (A-T) is a very rare multisystem disease of DNA repair, associated with progressive disabling neurological symptoms, respiratory failure, immunodeficiency and cancer predisposition, leading to premature death. There are no curative treatments available for A-T but clinical trials have begun. A major limiting factor in effectively evaluating therapies for A-T is the lack of suitable outcome measures and biomarkers. We have performed a systematic review to collect the information currently available on biomarkers for A-T both in patients and preclinical studies. We have identified 56 reports discussing potential A-T biomarkers in both pre-clinical models and patients. These studies report on diagnostic biomarkers but prognostic biomarkers and responsive markers of clinical status are currently lacking. Some biomarkers of neurodegeneration in A-T show promise, including non-invasive neuroimaging biomarkers. Some biomarkers of oxidative stress and responsive markers to radiotherapy and steroid treatment have potential value in clinical trials. The formation of the A-T biomarker working group with international experts is an important step forward to facilitate the sharing of materials, data and expertise with the common goal of finding effective biomarkers for A-T.

**Supplementary Information:**

The online version contains supplementary material available at 10.1007/s00415-024-12766-7.

## Introduction

Ataxia-Telangiectasia (A-T) (OMIM 208900) is a rare autosomal recessive DNA repair disorder associated with neurodegeneration, elevated malignancy risk, respiratory disease, immunodeficiency and metabolic disruptions [1]. The ataxia telangiectasia-mutated (*ATM*) gene encodes the ATM protein, a serine-threonine kinase mainly involved in the response to DNA double-strand breaks and oxidative stress [2]. Patients with a complete lack of ATM kinase activity have “classic A-T” with characteristic disabling neurological symptoms of cerebellar ataxia, dysarthria, peripheral neuropathy, extrapyramidal features and oculomotor apraxia [3]. Most patients are wheelchair-dependent by the time they begin their second decade of life and have a markedly reduced average life expectancy to their mid-twenties due to malignancy, lung failure, or infection [4]. Diagnosis of A-T currently relies on clinical correlation by experienced clinicians and ordering the correct exome sequencing panels. Patients with residual ATM kinase activity have milder disease, the so-called “variant A-T”, generally without respiratory insufficiency or immunodeficiency. Variant A-T patients have a diverse neurological phenotype, often leading to delayed diagnosis of sometimes up to 20 years [5].

A-T causes significant morbidity and there is no known cure and only supportive, non-specific treatments are available to date. With an incidence rate between 1:40,000 to 1:100,000 [6], the scope for developing large-scale studies is challenging. In the era of widely available exome sequencing and newborn screening for immunodeficiencies, there is a need for reliable diagnostic biomarkers to prove or disapprove pathogenetic of ATM variants, and to predict the course of the disease. Previous clinical treatment studies have mainly focused on clinical severity scores such as the Scale for Assessing and Rating Ataxia (SARA), International Cooperative Ataxia Rating Scale (ICARS) [7] or the AT-NEST score [8]. Although severity scores are useful for rapid clinical correlation, suitably trained investigators are required to perform the clinical examination to ensure reproducibility. Mild improvements in clinical severity scores may reflect day-to-day symptom fluctuation. Disease progression biomarkers would serve as an unbiased objective measure to evaluate treatment effectiveness but they are currently lacking.

As treatment trials for A-T are underway, the employment of diagnostic and disease progression biomarkers is now imperative. To that end, as part of an international collaborative effort to find potential diagnostic and clinical trial-appropriate biomarkers for A-T, we have conducted a systematic review of the existing literature for biomarkers of neurodegeneration, immunological abnormalities and radiation sensitivity in A-T patients and in cell culture models.

## Methods

### Systematic review protocol

We aimed to identify specific diagnostic and prognostic biomarkers for A-T. The parameters for the systematic review were defined by the A-T International Working Group meeting and registered with the International Prospective Register of Systematic Reviews (PROSPERO) (CRD42024536897). The systematic review is reported following the guidelines set out by the Preferred Reporting Items for Systematic Reviews and Meta-Analyses (PRISMA) Statement. (https://doi.org/10.1371/journal.pmed.1000097).

### Literature search strategy

To include all existing literature on biomarkers in A-T, we searched PubMed using a combination of free words and controlled vocabulary terms—(ataxia telangiectasia OR ATM OR AT OR Louis-Bar syndrome) AND (biomarker OR surrogate marker OR marker) on 25th August 2022. We restricted the time frame to 1995 to present, as the ATM mutation underlying A-T was first reported and described in 1995 (https://doi.org/10.1126/science.7792600).

## Results

**Fig. 1:**
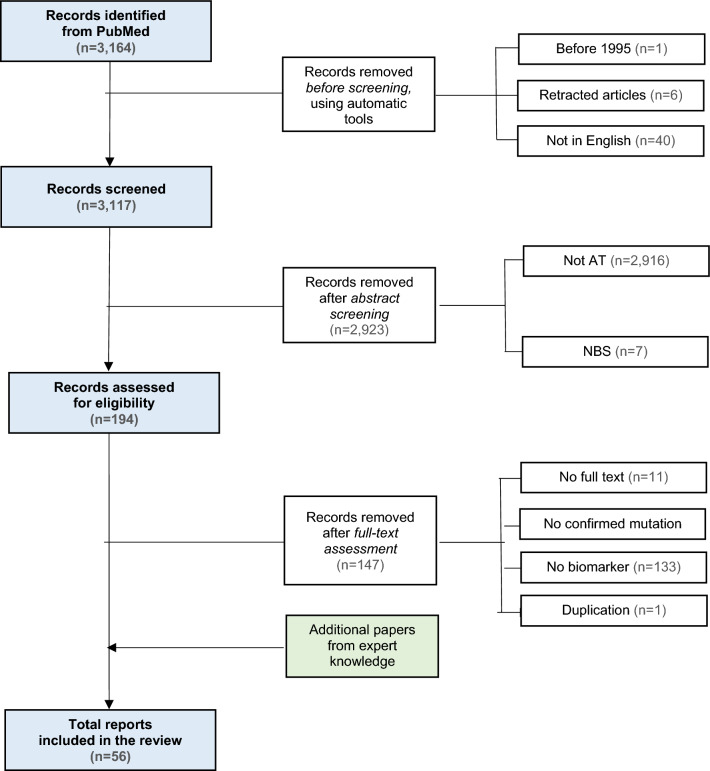
PRISMA flow diagram of articles screened for biomarkers in Ataxia Telangiectasia

**Fig. 2:**
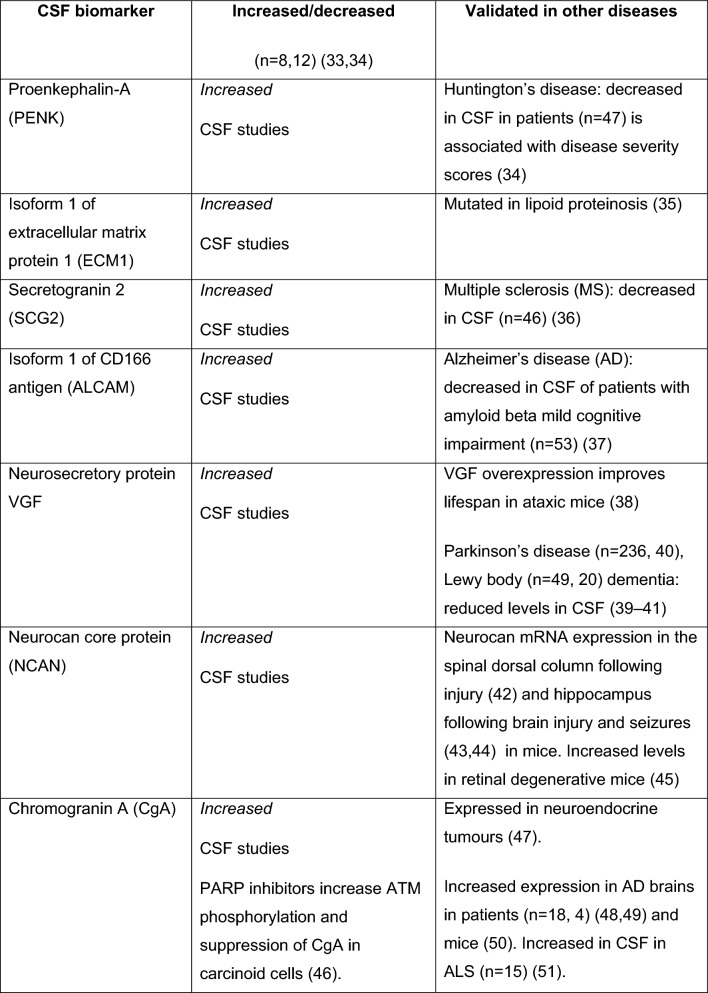

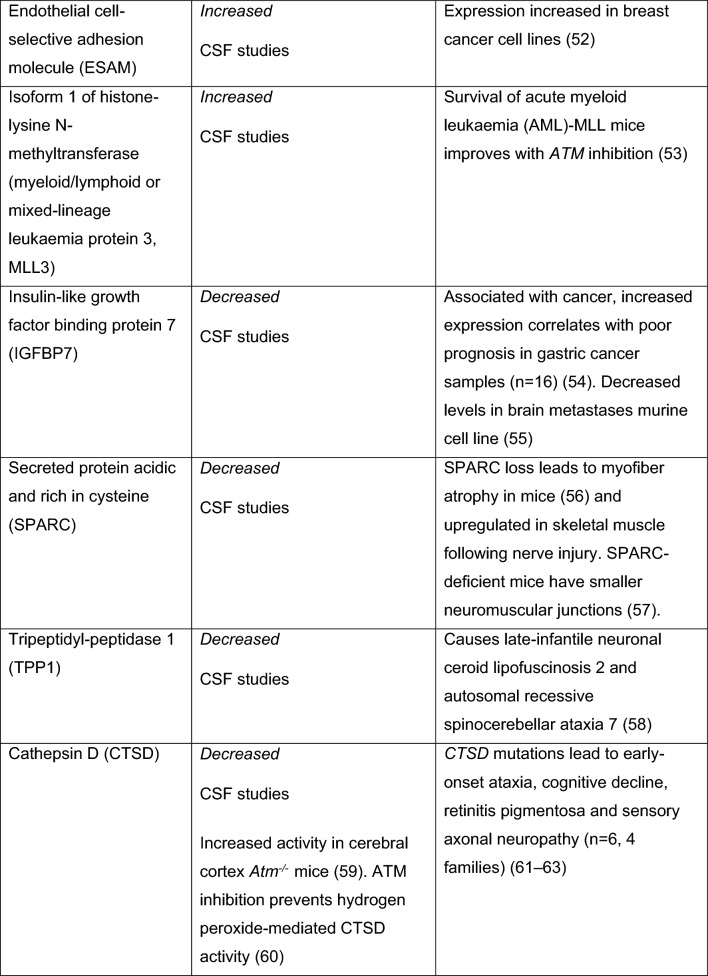

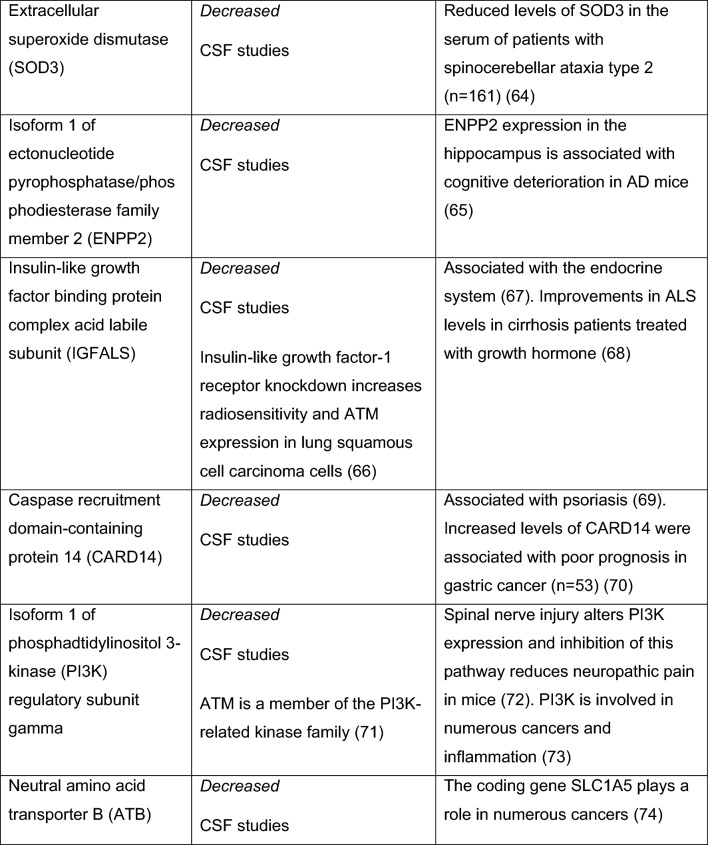
CSF protein biomarkers in A-T, many associated with neurodegeneration and cancer. Key: *AD* Alzheimer’s disease, *ALS* amyotrophic lateral sclerosis, *PD* Parkinson’s disease

**Fig. 3:**
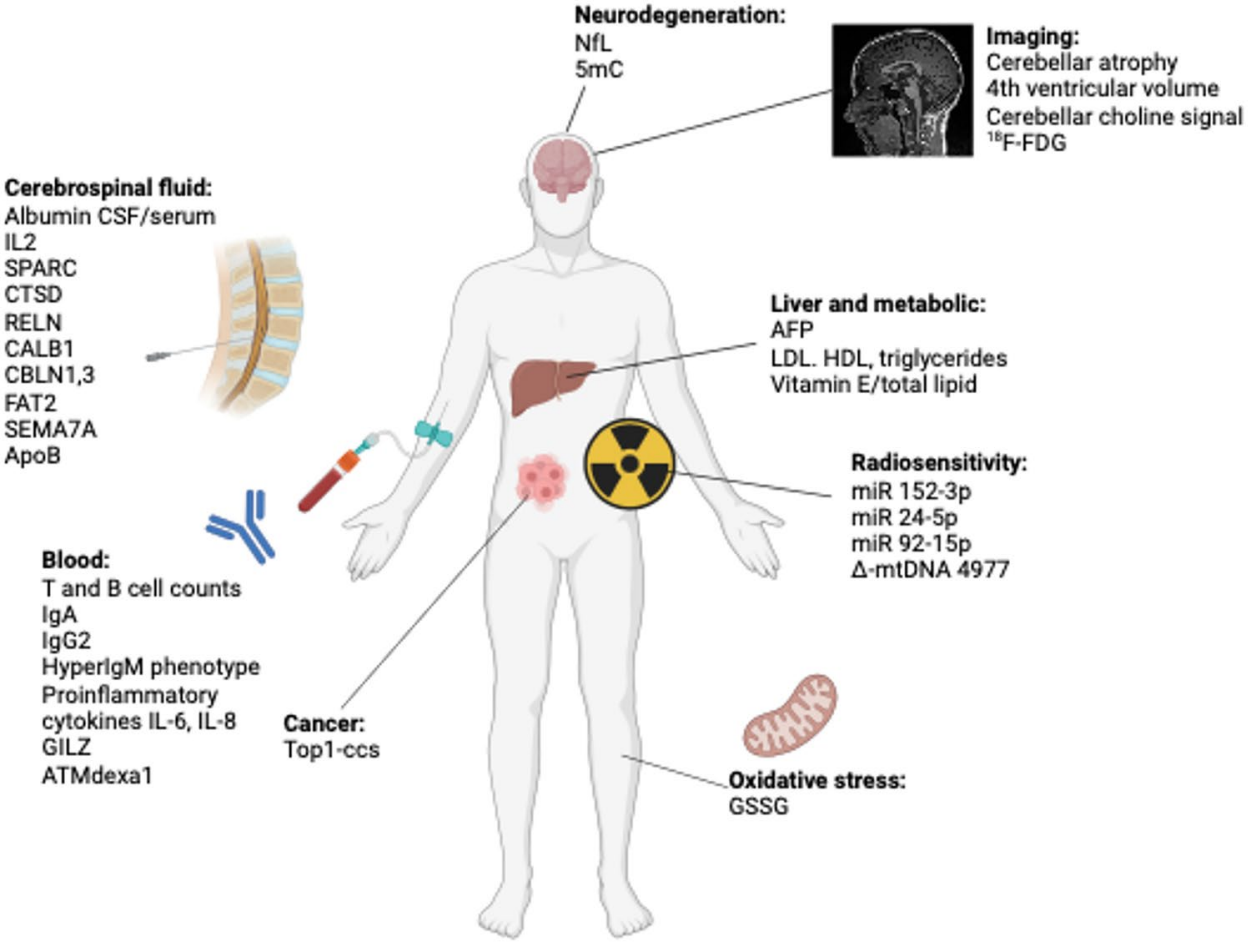
A-T biomarkers from patient studies. *NfL* neurofilament light chain, *5mC* 5-hydroxymethylcytosine, ^*18*^*F-FDG* ^18^F-fluorodeoxyglucose, *AFP* alpha fetoprotein, *LDL* low density lipoprotein, *HDL* high density lipoprotein, *miR* miRNA, *mtDNA* mitochondrial DNA, *GSSG* glutathione, *Top1-css* topoisomerase 1-DNA covalent complexes, *GILZ* glucocorticoid-induced leucine zipper, *IL2* interleukin2, *SPARC* secreted protein acidic and rich in cysteine, *CTSD* cathepsin D, *RELN* reelin, *CALB1* calbindin, *CBLN* cerebellin, *FAT2* protocadherin fat 2, *SEMA7A* semaphorin 7A, *ApoB* Apolipoprotein B

**Fig. 4:**
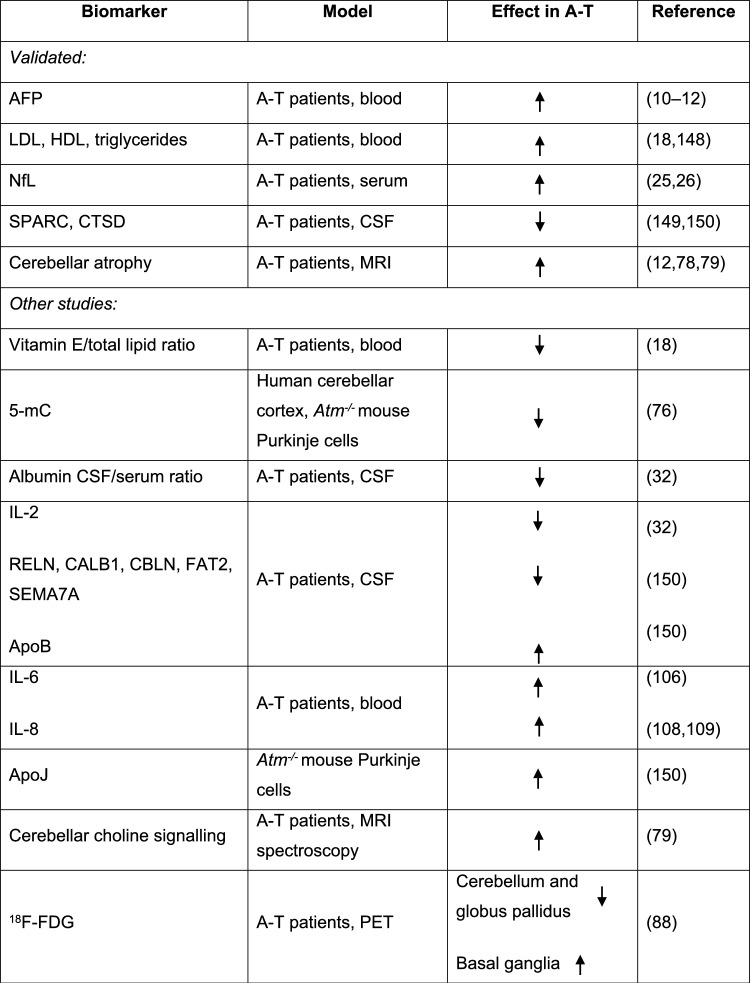

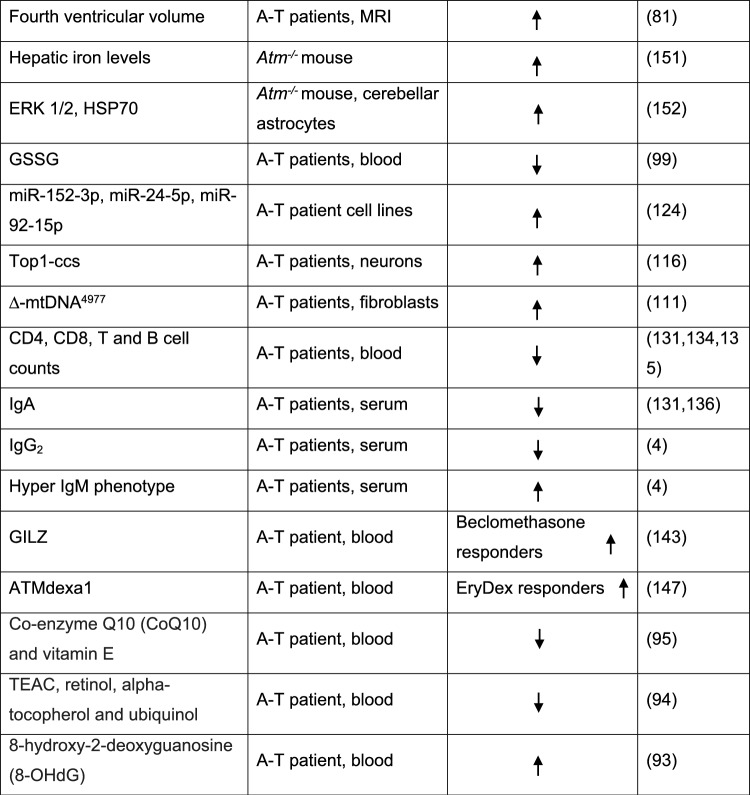
Summary of the relevant A-T biomarker studies. *NfL* neurofilament light chain, *5mC* 5-hydroxymethylcytosine, ^*18*^*F-FDG* ^18^F-fluorodeoxyglucose, *AFP* alpha fetoprotein, *LDL* low density lipoprotein, *HDL* high density lipoprotein, *miR* miRNA, *mtDNA* mitochondrial DNA, *ERK* extracellular signal-regulated protein kinase, *HSP70* heat shock protein 70, *GSSG* glutathione, *Top1-css* topoisomerase 1-DNA covalent complexes, *GILZ* glucocorticoid-induced leucine zipper, *EryDex* intraerythrocyte dexamethasone, *IL2* interleukin2, *SPARC* secreted protein acidic and rich in cysteine, *CTSD* cathepsin D, *RELN* reelin, *CALB1* calbindin, *CBLN* cerebellin, *FAT2* protocadherin fat 2, *SEMA7A* semaphorin 7A, *ApoB* Apolipoprotein B, *CoQ10* coenzyme Q10, *TEAC* total antioxidant capacity, *8-OHdG* 8-hydroxy-2-deoxyguanosine

A total of 3,164 reports were found and uploaded onto the Rayyan QCRI platform. A full search strategy including search terms and filters can be found in Supplementary File 1. We excluded 47 articles including retracted articles, and articles not written in English or published before 1995 (Fig. 1, 2, 3, 4).

### Inclusion and exclusion criteria for screening

For the remaining reports, both the title & abstract and the full text were screened by two independent blinded reviewers (BG and MYT) and arbitrated by an expert reviewer (RH). We implemented the following inclusion and exclusion criteria (Text box 1).
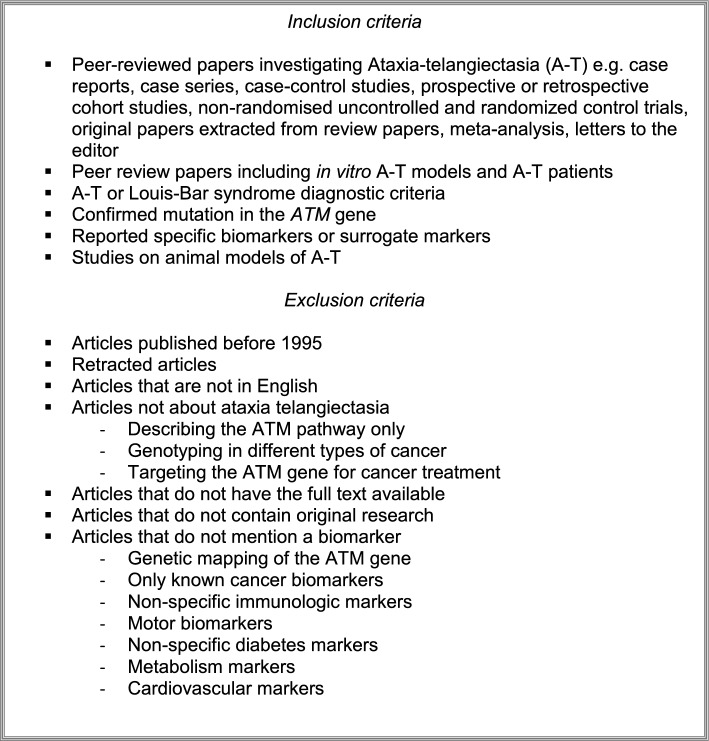


### Data extraction

A total of 56 reports met our inclusion criteria. The complete articles were grouped based on biomarker subtype, in vitro model, patient studies or interventional clinical trials. Full data extraction can be found in Supplementary File 2. Where possible, the demographic data and effect size have been included, such as fold change, mean, standard deviation, or range, if discussed in the original manuscript.

Evaluation of biomarkers.

### Serum alpha fetoprotein (AFP)

Alpha Fetoprotein (AFP) is a foetal protein that is still present in serum in high concentrations at birth and slowly decreases to normal’’adult’’ levels around the age of two years [9]. In patients with A-T, serum AFP levels usually remain elevated [10, 11]. The precise mechanism for the failed decrease in serum AFP levels in patients with A-T is unknown. Although AFP is considered a reliable diagnostic biomarker for the disease, in a small proportion of variant A-T patients, AFP can be normal [12]. Furthermore, there is no clear association of AFP levels with disease severity [13], making this biomarker not suitable for therapeutic trials.

### Lipoproteins

Altered lipid metabolism has been implicated in neurodegenerative disorders such as Parkinson’s disease, Alzheimer's disease and multiple sclerosis [14–16] but the mechanism is unclear. Impaired lipid homeostasis can potentially lead to impaired mitochondrial oxidative phosphorylation [17]. Metabolic syndromes are frequently associated with classic A-T. Commonly, triglyceride and low-density lipoprotein (LDL) levels are elevated with lower high-density lipoprotein (HDL) and lower vitamin E (per total lipid ratios) [18]. We also note associated fatty liver disease and type 2 diabetes which is progressive with age [19, 20]. Such findings are not specific to A-T, ataxia with oculomotor apraxia type 1 (AOA1) is also associated with elevated lipid levels [21]. The correlation between lipoprotein levels and clinical presentation of A-T has not been elucidated.

### Biomarkers for neurodegeneration

Neurological symptoms in A-T are present in both classic and variant A-T and lead to considerable morbidity. The neurological phenotype has not been recapitulated in animal models with ATM mutations but biomarkers for neurodegeneration derived from patient samples and cell lines are emerging.

Neurofilament light chain (NfL) has gathered increasing interest in A-T and other neurodegenerative diseases such as Hereditary Motor and Sensory Neuropathy (Charcot-Marie-Tooth disease), multiple sclerosis and Parkinson’s disease, suggesting that elevated levels in the blood and cerebrospinal fluid (CSF) are associated with axonal damage [22–24]. Serum and plasma NfL are increased in A-T compared to age-matched controls and higher in classic than variant A-T, suggesting that NfL may serve as an indicator of disease severity [25, 26]. NfL reduces with age in A-T patients [27] and not specific to A-T, therefore, further longitudinal studies on the clinical relevance of NfL as a molecular biomarker in A-T are needed. NfL levels did not change in a 24-month longitudinal study of nicotinamide riboside (NR) therapy. During this study, NR was associated with improvement in disease severity scores (SARA, AT-NEST, ICARS) and oculomotor function [27]. NfL has been assessed as a longitudinal biomarker in a placebo-controlled randomized trial of triheptanoin (NCT04513002). The results of this study are awaited.

NfL has gathered increasing interest in other inherited ataxias, such as spinocerebellar ataxia type 3 (SCA3) [28, 29]. Similar to A-T [27], NfL levels are increased in SCA3, the difference is greater in younger patients [28]. NfL was elevated in pre-ataxic patients and early disease and correlated with SARA score [28, 29]. NfL levels are increased in Friedreich ataxia but does not correlate with disease severity [30]. In summary, whilst NfL levels are increased in A-T, similar to other neurodegenerative diseases, its usefulness as a marker of disease severity in interventional trials is as yet not established and further longitudinal studies are required.

### CSF biomarkers

CSF is a filtrate from the choroid plexus in the brain. The proteins present in CSF, usually obtained by lumbar puncture (LP), contain important diagnostic information for many neurological disorders [31]. Albumin CSF/serum ratio (AR) is significantly increased with age in A-T, indicating the disruption of the blood–brain barrier. Inflammatory cytokines are not elevated, but IL-2 is decreased in the CSF and post-LP complications have been observed more commonly in A-T patients than in other diseases [32]. Although none of the aforementioned CSF proteins were validated in other tissues, such as fibroblasts [33].

### Cytosine modification biomarkers

Cytosine modification is associated with tumorigenesis. Of the 5’-CpG-3’ dinucleosides, 5-hydroxymethylcytosine (5mC) is one of the most abundant epigenetic biomarkers [75]. 5-mC is found in high levels in neurons and is a potential cancer biomarker. In patient and *Atm*^*−/−*^ mouse Purkinje cells, 5mC levels are reduced, leading to alteration of chromatin structure due to unknown mechanisms [76]. Cancer-specific 5-mC patterns are seen in the cell-free DNA of cancer patients (colorectal, gastric, pancreatic, liver, thyroid) [77]. Further studies distinguishing 5mC levels from other cancer patients without ATM defects are required.

### Neuroimaging biomarkers

The most common brain MRI finding during diagnostic workup for A-T is cerebellar atrophy [12, 78, 79], but this may not be detectable in very young children at the time of first presentation. Quantitative studies have shown reduced volume of the cerebellar vermis and hemispheres compared to controls [80] which progresses through childhood [81]. Cerebellar atrophy is not specific for A-T, but a number of studies have reported the finding of cerebral hypointense foci on susceptibility-weighted imaging (SWI) in A-T [79, 82–85], and the combination of cerebellar atrophy and cerebral SWI hypointense foci in a child or young person with ataxia has potential as a specific diagnostic biomarker for A-T warranting further investigation.

Magnetic resonance spectroscopy (MRS) allows quantification of brain tissue metabolites. MRS studies in both children and adults with A-T have shown reductions in cerebellar N-acetyl aspartate (NAA) compared to healthy controls, expressed as normalized NAA, NAA to creatine ratio and NAA to choline ratio [79, 81, 86]. NAA is a marker of neuronal health, and the reduction in the cerebellum of people with A-T is likely to reflect neurodegeneration. Studies have also identified increased ratios of cerebellar choline to creatine [79, 81], which is likely to be a marker reflecting myelin turnover.

Diffusion MRI allows quantification of ultrastructural barriers to water motion and provides in-vivo measures of tissue cellularity and white matter integrity. Studies in A-T are limited, but Sahama et al*.* used diffusion tensor imaging (DTI) to quantify reduced tract integrity at multiple sites in the motor pathways of children and young adults with A-T compared to controls. The findings suggested alterations in the corticospinal and somatosensory tracts and in the cerebellar-thalamo-cortical pathway [87]. Diffusion MRI studies of intracerebellar white matter have shown loss of ultrastructural white matter integrity [80, 81].

^18^F-fluorodeoxyglucose (^18^F-FDG) positron emission tomography (PET) provides a marker of brain glucose metabolism. PET studies are limited in A-T, but Volkow and colleagues demonstrated reduced uptake in the cerebellum, fusiform gyrus and hippocampus and increased uptake in the globus pallidus of people with A-T compared to controls [88].

In a regression analysis of possible quantitative imaging predictors, fourth ventricular volume was the only variable that predicted neurological status as determined by the A-T Neurological Examination Toolkit (A-T-NEST) [81], Uptake of ^18^F-FDG in the globus pallidus of people with A-T has been shown to correlate negatively with motor performance [88]. While these imaging biomarkers have the potential to be clinically useful in A-T, they currently lack independent validation and require evaluation in longitudinal natural history studies with larger cohorts of patients.

### Lung function parameters

Lung disease is a prominent characteristic and significant prognostic factor of A-T. Spirometry is an important diagnostic tool for the detection of lung disease. In many previous studies, lung function tests were performed on A-T patients. Repeatedly, A-T was shown to be associated with a progressive decrease in FVC and FEV1 [89–92]. A rapid increase in the FEF25-75/FVC ratio was associated with mortality within 2–3 years in a retrospective work [91]. However, forced spirometry results are influenced by a combination of lung volume, airway caliber, muscle power, neurological co-ordination of breathing, and the ability to make a seal around a mouthpiece. Low spirometry can be a result of any of these processes, and work is ongoing for biomarkers that more accurately assess individual components of lung function in A-T.

### Oxidative stress

Reichenbach et al*.* demonstrated increased levels of lipid peroxidation products and oxidative stress-related DNA bases, such as 8-hydroxy-2-deoxyguanosine (8-OHdG) as well as decreased plasma total antioxidant capacity (TEAC), retinol, alpha-tocopherol and ubiquinol in A-T patients’ peripheral blood [93, 94]. Pietrucha et al*.* showed increased total oxidant status (TOS) and oxidative stress index (OSI) and reduced total antioxidant status (TAS), coenzyme Q10 (CoQ10) and vitamin E, respectively [95].

ATM is activated by increased production of reactive oxygen species from oxidative and metabolic stress [96] in conditions of glucose depletion, calcium transfer and when transfer between the endoplasmic reticulum and mitochondria is impaired [97, 98]. In A-T patients, blood glutathione levels (GSSG) were decreased suggesting adaptations to oxidative stress [99, 100].

Loss of ATM function leads to ROS accumulation and oxidative stress [101]. Nuclear factor erythroid 2-related factor (Nrf2) is a transcription factor that protects cells from oxidative stress and interacts with ATM- and Rad3-related protein (ATR) in response to double-stranded DNA breaks (DSBs) [102]. In Friedreich ataxia, the oxidative stress response is impaired due to the failure of NRF activation [103]. Treatment with Nrf2 activator, Omaveloxolone, restores complex I activity and protected from oxidative stress in mouse models and human primary fibroblasts. Omaveloxolone improves neurological symptoms in Friedreich ataxia patients [104]. In A-T, Nrf2 protein levels are not increased but nuclear translocation of NRF2 is promoted by Dexamethasone treatment in patient fibroblasts [105]. Further studies into the feasibility of Nrf2 as a progressive biomarker and therapeutic target in A-T are required.

Proinflammatory cytokines have been investigated in A-T patients, notably IL-6 and IL-8 are elevated in A-T patients, suggesting a chronic inflammatory state [100, 106–109]. In A-T fibroblasts, glutamine deprivation triggers the production of ROS and IL-8 [110] and is inhibited with anti-oxidant alpha lipoic acid [107]. Elevated IL-8 levels are associated with increased malignancy risk and mortality in a follow-up study and reanalysis by McGrath-Morrow et al*.* [109].

### Biomarkers related to mitochondrial dysfunction

Mitochondrial DNA (mtDNA) accumulation or depletion can be an indicator of mitochondrial degeneration and neurodegenerative disorders. The accumulation of a common deletion (Δ-mtDNA^4977^) can be used as a surrogate marker for total mtDNA damage. Radiation exposure to immortalised radiosensitive A-T fibroblasts leads to the accumulation of Δ-mtDNA^4977^, raising the possibility that mtDNA abnormalities may contribute to the neurodegeneration in A-T [111]. The role of mitochondria and DNA repair in A-T is unclear, however, it is thought that the fusion of mitochondria from healthy cells is facilitated by ATM [112].

### Radiosensitivity and cancer biomarkers

Cancer predisposition in A-T (irrespective of neurological disease severity) and heterozygous carriers highlight the importance of early diagnosis. A Delphi-based consensus survey of 35 panelists from 6 continents (Europe, Africa, Asia, Oceania, North and South America) support the need for evidence-based guidelines for cancer surveillance [113]. Currently, there are no universally agreed guidelines for cancer screening in A-T, although a recently completed feasibility trial (NCT05252819) of whole-body MRI cancer surveillance showed the approach is feasible and well-accepted by children and young people with A-T and their families [114].

Tumour suppressor gene p53 is vital in DNA repair responses. A-T fibroblasts showed lower induction of p53 and p21 and their phosphorylated substrates after irradiation compared to fibroblasts from breast cancer patients, A-T heterozygotes [115] and controls [116]. The clinical significance of this is unknown and does not explain why heterozygous carriers are also at increased risk of cancer [115]. The reduced level of phosphorylated p53 has also been reported in A-T-like disorder (ATLD) [128].

Radiosensitivity is a hallmark of DNA repair disorders such as A-T. Radiotherapy is a common treatment for cancers but can lead to severe reactions in A-T patients [117] but the degree of radiosensitivity is variable. Early detection of radiosensitivity can be an effective way to assist in diagnosis and avoid exposure to radiation from investigations and cancer treatment. Currently, radiosensitivity is determined using chromosomal breakage studies and chromosomal rearrangements after irradiation [118] in specialist diagnostic laboratories.

Histone H2A.X is phosphorylated by ATM during DSB repair, creating γ-H2A.X, and has previously created interest as a potential radiosensitivity biomarker for A-T. The results so far for γ-H2A.X have been mixed or have utilized a small number of A-T patient samples [116, 119–123]. Bryant et al*.* reported unchanged γ-H2A.X levels in all A-T cell lines but suggest that testing microRNA (miR-152-3p, miR-24-5p, miR-92-15p) associated with modulation of tumor suppressor phosphatase and tensin homolog (*PTEN*) and cyclin D1 (*CCND1*) may be of interest [124].

High levels of chromosomal abnormalities occur in about 10% of lymphocytes in A-T patients and can assist diagnosis. Most common is the t(7;14) translocation [125], estimated to be increased by 40-fold in A-T patients [126]. This is not a specific diagnostic biomarker as the translocation is also present in Nijmegen Breakage Syndrome (NBS) [127]. Elevated levels of complex aberrations and dicentric chromosomes have been detected after irradiation in A-T [128]. Colony survival assays (CSA) have previously been evaluated as an adjuvant biomarker for radiosensitivity but has a long turnaround time which would not be practical in the initiation of treatment of A-T patients with cancer [129]. Conversely, Bcl-2 apoptotic regulatory proteins, do not correlate with radiosensitivity in A-T cell lines [130].

### Immunodeficiency

Most classic A-T patients show variable signs of immunodeficiency that, conversely, are uncommon in patients with variant A-T [5, 131]. Cases of A-T have been diagnosed using the T cell receptor excision circle (TREC)-base newborn screening [132, 133]. Reduced T- and B-cell counts, principally naïve CD4^+^ T, CD8^+^ T and B cell numbers, are found in almost all classic A-T patients [131, 134]. Of note, in an Italian cohort of 66 A-T patients, lymphopenia at diagnosis was associated with earlier age at disease onset and reduced life expectancy [135].

Deficiency of humoral immunity in A-T is more variable, including decreased or absent serum immunoglobulin A (IgA), IgG_2_, or IgE, increased serum IgM levels, and impairment of antibody responses to a variety of microbial antigens [131, 136]. In one retrospective study on 61 A-T patients, selective deficiency of IgG_2_ and the hyper IgM phenotype correlated with reduced survival [4]. Immunodeficiency in these patients may become clinically apparent over time due to progressive ageing of the immune system in the context of reduced ATM kinase activity [137].

Ineffective immune responses against viruses and cancer may contribute to A-T disease progression and reduced survival. Monitoring of immunoglobulin levels to characterize individual immunodeficiency profiles and prevent risk of infection is recommended [138].

### Biomarkers used in clinical trials with Steroids in AT

The use of steroids in A-T for improvement in neurological symptoms is not universally accepted in A-T patients. Steroids lead to increased longevity and reduced microglial activation in a rat model of A-T with spinal cord atrophy [139]. Dexamethasone therapy improves nuclear translocation of NRF2 in A-T fibroblasts and lymphoblastoid cell lines [105, 140].

Transient improvements in patient SARA scores with short-term steroid use have been reported [141, 142] with deterioration during steroid washout periods [143]. Glucocorticoid-induced leucine zipper (GILZ) levels correlated with lower dose oral beclomethasone use and improved SARA scores. Following a second cycle of higher-dose beclomethasone, GILZ levels were not elevated in all patients [143].

In an attempt to ameliorate the long-term side effects of oral steroid use, autologous intra-erythrocyte dexamethasone (EryDex) allows for a slow delivery of encapsulated dexamethasone and has been explored with reported neurological symptomatic improvement measured by the ICARS score [144, 145]. The aim of the ATTeST study was to evaluate the efficacy and safety of intra-erythrocyte delivery of dexamethasone compared with placebo in children with ataxia telangiectasia [146]. Although there were no safety concerns, the primary efficacy endpoint was not met. Studies continue with EryDex in participants aged 6–9 years, on the basis of findings from subgroup analyses from this trial to define whether this treatment leads to the improvement in neurological symptoms (NCT02770807). ATMdexa1 transcript expression is higher in EryDex responders. As ATMdexa1 is undetectable in untreated A-T patients, the use of this biomarker would be reserved for the use of steroid therapy [147].

## Discussion

This systematic review identified 56 reports discussing potential A-T biomarkers in both pre-clinical models and patients. Many of the manuscripts discuss biomarkers to explore disease mechanisms, while some biomarkers have been described only in single manuscripts in pre-clinical models. This can lead to potential selection and chronological bias, particularly from studies involving patient registries or studies with a lack of case controls. Where possible, we have evaluated the effect size of each potential biomarker (Supplementary file 2), however, this was mainly limited to clinical studies and is not standardised as we do not have the original data for the published studies. Pre-clinical studies describe increased or decreased expression of biomarkers, which do not allow quantitative evaluation of the effect size. We provide a comprehensive list of potential biomarkers to date and their value in A-T. A small number of validated biomarkers are not unique to A-T, such as AFP and triglycerides.

Some biomarkers of neurodegeneration in A-T show promise, including non-invasive neuroimaging biomarkers. Cerebellar atrophy is a universal feature of A-T but is non-specific. Fourth ventricular volume [81] and characteristic ^13^F-FDG signatures [88] are potential novel biomarkers, which require further clinical validation with longitudinal studies.

In recent years, large-scale omics have been employed for CSF studies in A-T, with reproducible results. Some of the CSF biomarkers described are not novel to A-T but have been previously described in other neurodegenerative disorders, malignancies, endocrine and dermatological disorders. Though lacking specificity, unique CSF biomarkers for A-T should be evaluated in longitudinal studies against established neurodegenerative biomarkers, such as NfL. In combination with mechanistic biomarkers, such as NrF2, these biomarkers provide further insight into the complex multiorgan involvement of A-T. Such biomarkers are not necessarily useful for diagnostic purposes but have a potential in treatment trials.

There are potential biomarkers emerging for immunodeficiency, chronic inflammation (CD4^+^, CD8^+^, T cells, B cells, IgA, IgG_2_, hyper IgM, IL-6 and IL-8) and steroid therapy use. GILZ and ATMdexa1 have been identified as useful responsive biomarkers for steroid therapy. However, to date, steroids are not routinely prescribed to A-T patients. More studies are required to assess the effectiveness of long-term steroid therapy. The advent of EryDex may circumvent the potential side effects of traditional oral steroid use.

In the era of first-line genomics *ATM* variants are frequently being identified and diagnostic biomarkers (AFP, NfL, cellular and humoral immunodeficiency, 5-mC) can assist in the validation of pathogenicity. However specific prognostic biomarkers, enabling quantitative monitoring of clinical progression are still lacking in A-T.

As new omics technologies are becoming more and more available and have been applied to other genetic ataxias such as ARSACS [152] and Friedreich ataxia [153,154], there is a hope that novel molecular biomarkers will be identified also in A-T. As A-T has a diverse clinical phenotype, samples from a diverse range of patients are required to validate the sensitivity and specificity of novel biomarkers in A-T, therefore, the use of better in vitro models is still vital in A-T biomarker discovery. International collaboration and data sharing can facilitate novel biomarker discoveries, as shown in previous studies with NfL [25, 26]. The formation of the A-T biomarker working group with international experts on board for this very rare disease is an important step forward to facilitate the sharing of materials, data and expertise with the common goal of finding effective biomarkers for A-T.

## Supplementary Information

Below is the link to the electronic supplementary material.

Supplementary file1 (XLSX 9 kb)

Supplementary file2 (XLSX 20 kb)

## Data Availability

All available data has been included in the supplementary files and have been stated within the manuscript.
